# AI-enabled drug repurposing in Alzheimer’s disease: from computational nomination to pharmacological proof

**DOI:** 10.3389/fphar.2026.1999276

**Published:** 2026-10-28

**Authors:** Xinzhong Li, Illana Gozes

**Affiliations:** 1 School of Health and Life Sciences, Teesside University, Middlesbrough, United Kingdom; 2 The Elton Laboratory for Molecular Neuroendocrinology, Department of Human Genetics and Computational Medicine, Gray Faculty of Medical and Health Sciences, Sagol School of Neuroscience and Adams Super Center for Brain Studies, Tel Aviv University, Tel Aviv, Israel

**Keywords:** Alzheimer’s disease, artificial intelligence, clinical trials, drug repurposing, network medicine, neuropharmacology, pharmacoepidemiology, translational pharmacology

## Abstract

Artificial intelligence (AI) has expanded the scale and speed of drug repurposing in Alzheimer’s disease (AD). Transcriptomic signature matching, network-based systems pharmacology, real-world data pharmacoepidemiology and multi-omics machine learning have generated hundreds of candidate drugs, while repurposed agents account for 35% (56 of 158) of drugs and 42% (15 of 36) of Phase 3 agents in the 2026 AD pipeline. Clinical translation, however, remains unproven. Randomised trials of GLP-1 receptor agonists illustrate the gap between prioritisation and therapeutic proof: semaglutide did not slow clinical progression in evoke and evoke+, while liraglutide did not improve the primary FDG-PET endpoint in ELAD. We examine four complementary evidence domains, defining what each can and cannot establish, and position large language models as a cross-cutting tool for evidence synthesis and knowledge-graph reasoning rather than an independent source of biological evidence. Rather than cataloguing methods and candidates, we appraise leading drugs at the individual-molecule level and propose explicit criteria for progression from computational nomination to clinical testing. The framework defines seven requirements for clinical progression: disease-stage matching, adequate site-of-action exposure, including CNS exposure for central mechanisms, mechanistic validation in human-relevant models, biomarker-stratified trial design, convergence across independent evidence domains, causal inference with explicit boundary conditions, and mechanism-aligned endpoints with predefined stopping rules. AI has improved candidate prioritisation in AD, but clinical value will depend on converting ranked hypotheses into pharmacologically plausible and prospectively testable programmes.

## 1 Introduction

Alzheimer’s disease (AD) is the most common cause of dementia. An estimated 57.4 million people were living with dementia globally in 2019, and this number is projected to reach 152.8 million by 2050 (GBD, 2019, 2022). Against this burden, the therapeutic pipeline has historically been characterised by extreme attrition. A systematic review of interventional Phase II and III AD trials registered from 2004 onward identified 98 unique failed compounds against two successes at the time of that analysis (aducanumab and oligomannate), a Phase II/III success rate of 2.0% — approximately nine-fold below the 17.8% average across therapeutic areas, with development times some 40% longer (7.6 vs. 5.5 years) and costs more than twice as high ($5.69 billion vs. $2.56 billion) (Kim et al., 2022). The regulatory approvals of lecanemab in 2023 and donanemab in 2024 established that disease modification in early, biomarker-confirmed AD is achievable (van Dyck et al., 2023; Sims et al., 2023), while also defining the limits of current therapy. Both drugs are indicated only for early-stage disease with confirmed amyloid pathology; their use carries a risk of amyloid-related imaging abnormalities (ARIA), which is higher in apolipoprotein E ε4 (APOE4) carriers, and they impose substantial burdens related to infusion, monitoring and cost. Their clinical effects, although statistically significant, are modest.

Drug repurposing offers a structurally different proposition. Existing drugs already possess established human safety data, well-defined pharmacokinetic profiles, and established manufacturing processes; in principle, the time and cost required to advance from hypothesis generation to Phase 2 clinical trials represent only a fraction of what is needed to develop a novel molecular entity (Pushpakom et al., 2019). This logic has been embraced in the development of AD drugs. In the 2026 AD drug development pipeline, repurposed agents approved for non-AD indications account for 56 of 158 drugs in trials (35%) and 15 of 36 Phase 3 agents (42%) (Cummings et al., 2026a).

Artificial intelligence (AI) has been positioned as the engine that makes systematic repurposing tractable. Over the past decade, four families of computational method have been applied to AD: transcriptomic signature matching against perturbational drug databases; network-based systems pharmacology built on the human protein–protein interactome (PPI); pharmaco-epidemiological analysis of electronic health record (EHR) and claims data at population scale; and multi-omics integration using deep learning and graph neural networks (Cheng et al., 2024). More recently, large language models (LLMs) have also been added to this repertoire. Collectively these methods have produced an extensive candidate inventory. A systematic review of 124 AD repurposing studies published between 2012 and 2022 identified 573 unique drugs proposed as candidates (Grabowska et al., 2023). That inventory has not yet translated into clinical benefit. Of those 124 studies, 61% performed some form of validation, but only 4% (5 of 124) used real-world data. In our search through July 2026, we identified no computationally nominated repurposed drug that has demonstrated efficacy in a Phase 3 AD trial. The evoke and evoke+ trials provide the clearest recent test of the gap between observational nomination and clinical efficacy. In these trials, oral semaglutide showed no benefit over placebo on CDR-SB in 3,808 amyloid positive participants followed for 104 weeks, despite prior support from convergent pharmaco-epidemiological signals across independent databases and generative AI–based literature synthesis studies (Cummings et al., 2026b).

The difficulty facing AI-enabled repurposing in AD is no longer the generation of plausible candidates; it is the discipline required to distinguish computational nomination from translational evidence. Our central argument is that AI has improved candidate prioritisation in AD but has not yet demonstrated clinical success. Robust translation requires convergent evidence across computational, genetic, experimental, and clinical domains, together with adequate exposure and target engagement at the proposed site of action, population-level relevance, human-cell mechanistic validation, and biomarker-stratified clinical testing. We next define the strengths and limits of each AI evidence domain, evaluate leading candidates molecule by molecule against these criteria, and propose evidence standards for advancing candidates into clinical trials. Recent reviews have surveyed computational approaches, candidate drugs and broader opportunities for AI-enabled drug repurposing in AD (Cheng et al., 2024; Grabowska et al., 2023). Our focus is different: rather than expanding the candidate catalogue or comparing algorithms, we ask what evidence should be required after computational nomination. We therefore evaluate evidence at the individual-molecule level, distinguish nomination from independent validation, and define explicit translational requirements for progression to prospective clinical testing.

### 1.1 Review approach and candidate selection

This is a narrative rather than a systematic review, but we set out our search and selection procedure so that readers can judge what has been included and what has not. We searched PubMed, Embase and Web of Science, together with the ClinicalTrials.gov, EU Clinical Trials Register and WHO ICTRP registries, from January 2012 to the final search date of 31 July 2026. Search terms combined Alzheimer’s disease and dementia with drug repurposing, repositioning, artificial intelligence, machine learning, deep learning, network medicine, transcriptomic signature, large language model, target trial emulation and pharmacoepidemiology. Reference lists of retrieved reviews were hand-searched, and preprint servers (bioRxiv, medRxiv, Research Square) were checked for work not yet indexed.

We treated a candidate as computationally nominated if it was identified or prioritised by a documented computational procedure applied to biological or clinical data, including transcriptomic signature matching, network proximity analysis, machine learning classifiers, graph neural networks or large language model synthesis. We did not require that the method be described by its authors as artificial intelligence. Candidates arising solely from conventional pharmacology, clinical observation or investigator hypothesis were noted where relevant but were not treated as computational nominations.

Because 573 distinct agents have been proposed for repurposing in AD (Grabowska et al., 2023), detailed appraisal of every candidate is neither feasible nor informative. We therefore selected candidates for individual assessment if computational nomination formed a substantive part of the translational evidence chain and at least one independent validation domain was available, or if a clinically advanced non-computationally nominated programme provided a particularly informative test of the translational principles considered in this review. This yielded semaglutide and mirodenafil as clinically informative comparators, together with sildenafil, bumetanide, baricitinib and selected population- or LLM-supported candidates. Other repurposed agents with clinical data were outside the scope of the molecule-level appraisal; their omission should not be interpreted as a judgement on their therapeutic promise.

Trial status and negative findings were established from registry records and, where available, peer-reviewed publications rather than from press releases or conference abstracts alone. Because negative results are less consistently published than positive ones, we state negative claims as bounded by our search rather than as absolute: where we report that no study of a given kind exists, this should be read as meaning that we identified none up to 31 July 2026. The major computational and AI evidence domains used in AD drug repurposing, together with what each can and cannot establish, are summarized in Table 1.

**TABLE 1:** What each computational or AI evidence domain can and cannot establish in AD drug repurposing.

Evidence domain	What it establishes	What it cannot establish	Illustrative AD application
Transcriptomic signature matching	Pathway-level concordance between drug perturbation and disease signature; scalable across thousands of compounds; permits genotype stratification	That signature reversal confers clinical benefit; cell-type-specific effects from bulk tissue; relevance of cancer cell line perturbation data to CNS cells	DRIAD nomination of baricitinib from AMP-AD Braak-stage modelling (Rodriguez et al., 2021); APOE4-conditioned CMap query identifying bumetanide (Taubes et al., 2021)
Network-based systems pharmacology	Mechanistic plausibility grounded in human genetics and interactome topology; identification of polypharmacological candidates spanning endophenotypes	Target engagement in human brain; CNS exposure; disease-stage specificity; effects not represented in the mapped interactome, which remains incomplete	Endophenotype proximity nomination of sildenafil with claims-based validation (Fang et al., 2021); AlzGPS platform proximity scores for ∼3,000 drugs (Zhou et al., 2021)
Causal real-world evidence	Rate of incident dementia in drug-exposed versus comparator human populations, at scale, with real-world generalisability	Efficacy in established symptomatic disease; separation of prevention, metabolic mediation, prescribing bias, surveillance bias and residual confounding	Target trial emulation of semaglutide in 116M EHR records (Wang et al., 2024b); GLP-1RA and SGLT-2i versus DPP-4i comparisons (Zhang et al., 2025; Tang et al., 2025); discordant UK CPRD cohort (Wu et al., 2025)
Multi-omics integration and deep learning	Richer disease representation across molecular layers; cell-type context for candidate targets; improved ranking versus single-modality methods	Mechanistic interpretability sufficient to justify trial commitment; generalisation across cohorts with differing batch structure	Multiome frameworks integrating genome to phenome (Cheng et al., 2024); single-nucleus microglia–astrocyte network analysis (Xu et al., 2021)
Large language models (cross-cutting layer)	Rapid synthesis of published evidence; improved traversal of structured knowledge graphs relative to embedding methods; workflow orchestration	Any independent biological observation; mechanistic novelty beyond the training corpus; reproducibility across prompting strategies	GPT-4 candidate generation with EHR validation (Yan et al., 2024); ESCARGOT Graph-of-Thoughts reasoning over AlzKB (Wang et al., 2025); DeepDrug combination nomination (Li et al., 2025)

## 2 Alzheimer's disease is particularly suited to AI-enabled repurposing

AD has two characteristics that make it particularly suitable for computational drug repurposing: its polygenic, multi-pathway biology and the exceptional scale of available data. However, translation remains especially difficult because candidates acting through central mechanisms must achieve adequate exposure across the blood–brain barrier (BBB), a major translational challenge examined in Section 5.

### 2.1 Biological complexity and polygenic architecture

AD is not caused by a single pathway but involves at least six overlapping pathophysiological processes: amyloid deposition, tau aggregation, neuroinflammation, mitochondrial and metabolic dysfunction, cerebrovascular compromise and synaptic failure (Fang et al., 2020; Long and Holtzman, 2019). This architecture poses obstacles to single-target drug development, which has been the dominant paradigm and the dominant source of failure. It therefore favours approaches that can assess polypharmacology, in which a drug acts on several disease-related pathways at the same time. Network-based and multi-omics methods are designed for this purpose.

The 2026 pipeline reflects this diversification. It includes 192 trials of 158 drugs and spans 17 categories of AD biology, with immune, metabolic, vascular, synaptic and tau-directed strategies now developed alongside amyloid-directed programmes. Repurposed drugs account for 35% of all agents and 42% of Phase 3 agents (Cummings et al., 2026a). This breadth enlarges the space of approved compounds that may intersect AD biology, but it also increases the risk of weakly supported candidate generation. The value of AI lies less in producing a longer list than in identifying which molecules have sufficiently consistent evidence to justify further translational investment.

### 2.2 Rich molecular and clinical data resources

AD is among the most comprehensively profiled human diseases at population and molecular scale.

Human genetics provides an important anchor for computational drug repurposing, as disease-associated variants can help connect candidate targets and pathways to AD pathobiology. Genome-wide association studies (GWAS) have identified 75 loci associated with AD and related dementias, with strong enrichment for immune and lipid-processing pathways (Bellenguez et al., 2022; Kunkle et al., 2019). These loci supply the seed genes from which network-based methods construct disease modules, and they provide the single most useful filter for distinguishing mechanistically plausible from merely statistically associated candidates. Websites such as https://www.alzforum.org/ provide curated information on genetic variants, risk loci and candidate genes identified through AD GWAS and other genetic studies.

Transcriptomic resources are extensive and provide a complementary view of AD pathobiology by capturing disease-associated changes in gene expression across brain regions, cell types and disease stages. The Gene Expression Omnibus (GEO https://ncbi.nlm.nih.gov/geo/) is a public repository for high-throughput functional genomics data, including microarray and RNA-sequencing datasets. In addition, the Accelerating Medicines Partnership–Alzheimer’s Disease (AMP-AD) consortium has generated multi-omic post-mortem brain profiles across Braak stages from the Religious Orders Study and Memory and Aging Project, the Mayo Clinic Brain Bank and the Mount Sinai Brain Bank. Single-nucleus RNA sequencing has extended this to cell-type resolution, distinguishing disease-associated microglial states, reactive astrocyte phenotypes and selectively vulnerable neuronal populations (Xu et al., 2021). Spatial transcriptomics now maps expression to cortical layers and to plaque-adjacent microenvironments (He et al., 2024). These rapidly expanding datasets are increasingly organised through specialised resources such as the single-cell and spatial RNA-seq database for AD (ssREAD; https://bmblx.bmi.osumc.edu/ssread/). ssREAD integrates AD-related single-cell, single-nucleus and spatial transcriptomic datasets from human and mouse brains, together with information on brain region, disease status, sex, age and Braak stage (Wang C. et al., 2024).

Real-world clinical data are available at a scale unmatched in most disease areas. Optum Clinformatics (https://atlaslongitudinaldatasets.ac.uk/datasets/cdm), TriNetX (https://trinetx.com/), the All of Us Research Program (https://allofus.nih.gov/), Medicare claims and comparable national registries collectively cover well over 100 million individuals with longitudinal prescription and diagnostic records. This is what makes population-scale pharmacoepidemiology of dementia incidence feasible at all.

Integrated platforms have been built specifically to combine these layers for repurposing. AlzGPS (https://alzgps.lerner.ccf.org/) assembles more than 100 AD multi-omics datasets with the human protein–protein interactome (PPI) and approximately 3,000 approved and investigational drugs and makes network proximity scores publicly queryable (Zhou et al., 2021).

Therefore, from a clinical perspective, our challenge is not only to generate candidates for AD, but to identify those supported by sufficiently robust biological, pharmacological and clinical evidence to justify experimental validation and clinical testing.

## 3 Computational nomination and independent evidence sources

We consider four principal computational and evidence streams in AD repurposing: transcriptomic signature matching, network-based systems pharmacology, causal real-world evidence, and multi-omics machine learning. These are not all AI methods in a strict sense. Transcriptomic signature matching and network proximity analysis are bioinformatic and systems-pharmacology methods that may or may not employ machine learning, while target-trial emulation, propensity weighting and active-comparator designs are causal-inference methods drawn from epidemiology. We group them here because each contributes a distinct and non-interchangeable form of evidence about a candidate, not because each is an AI method. Experimental and pharmacological validation are treated separately as translational requirements in Section 5. Each stream answers a different question and has characteristic limitations.

Large language models (LLM) and knowledge-graph reasoning occupy a different position in this taxonomy. They retrieve, synthesise and reason over existing evidence; they do not generate a new class of biological observation. We therefore treat them as a cross-cutting methodological layer operating across the four domains, rather than as a fifth pillar of equivalent standing. This distinction is not merely taxonomic. Treating LLM output as an independent evidence stream risks double-counting the literature that produced it, and we return to this point in Section 3.5. Figure 1 summarises how these evidence sources should converge before a candidate is advanced.

**FIGURE 1:**
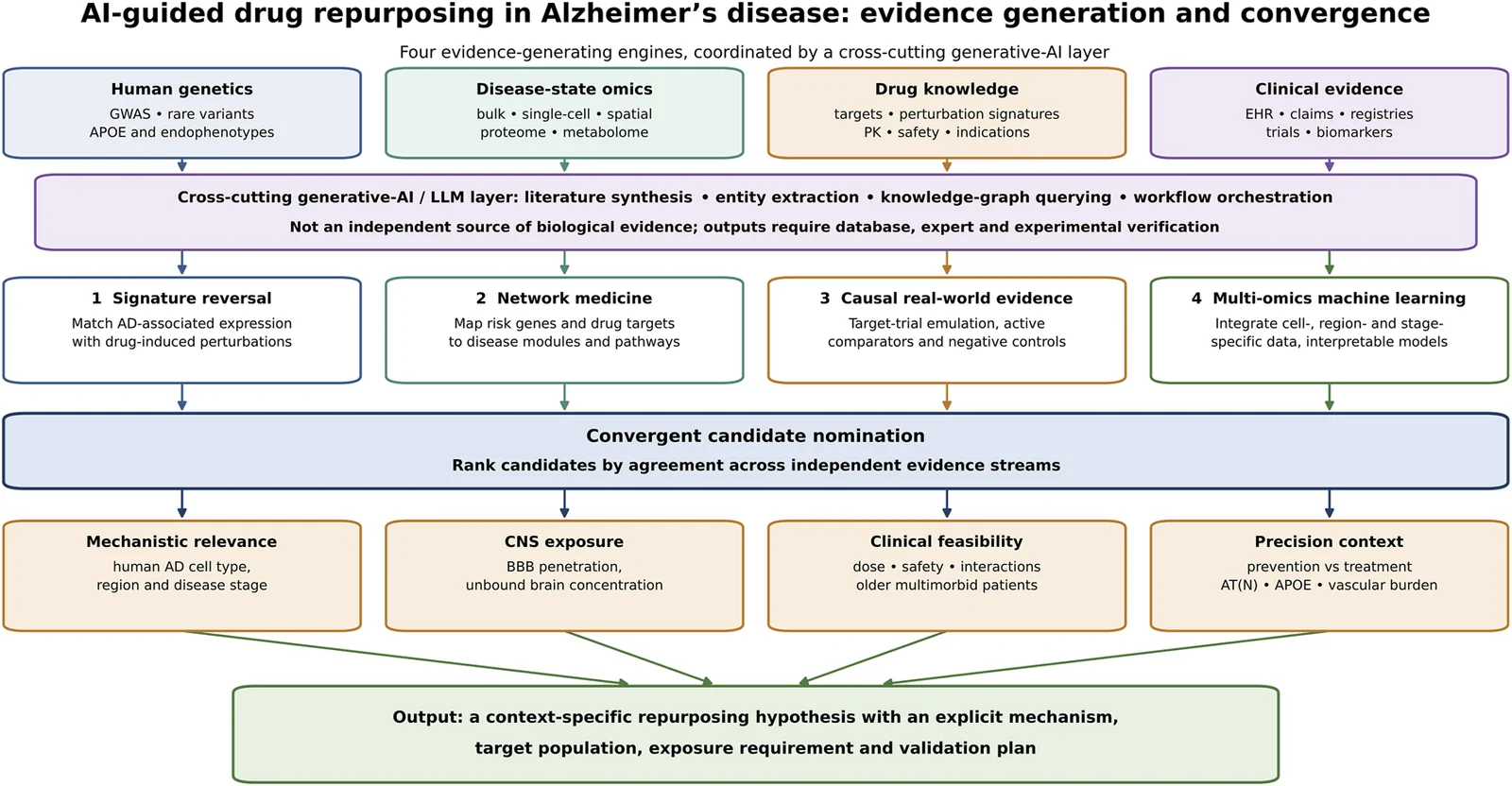
AI-guided drug repurposing in AD requires convergence across independent evidence streams. Human genetics, disease-state omics, drug knowledge and clinical data support transcriptomic signature reversal, network medicine, causal real-world evidence and multi-omics machine learning. LLMs operate across these domains for synthesis and workflow orchestration, but do not constitute independent biological evidence. Candidates should then be filtered for human disease relevance, site-of-action exposure, clinical feasibility and precision context before a testable translational hypothesis is defined.

### 3.1 Transcriptomic signature matching

The founding idea is straightforward: if a disease is characterised by a reproducible transcriptomic signature, compounds whose perturbational signatures are anticorrelated with it may reverse the disease state. The Connectivity Map (CMAP) established this paradigm (Lamb et al., 2006), and L1000CDS2 subsequently provided a search engine for querying characteristic-direction signatures derived from the LINCS L1000 resource (Duan et al., 2016).

Two AD applications are particularly instructive, as they illustrate complementary computational strategies for identifying and prioritizing repurposing candidates. The DRIAD framework trained a machine learning (ML) model to predict Braak stage from AMP-AD brain gene expression, then scored 80 approved drugs by the extent to which their target-associated gene sets shifted predicted pathology (Rodriguez et al., 2021). Janus kinase (JAK) inhibitors ranked highly, and baricitinib was carried forward into a biomarker-driven basket trial. Separately, Taubes and colleagues queried the CMAP with APOE genotype-specific signatures derived from human brain and induced pluripotent stem cell (iPSC)-derived neurons, identified bumetanide as a top signature-reversing candidate, and then validated it in APOE4 knock-in mice and in two independent EHR databases covering more than five million individuals (Taubes et al., 2021). The bumetanide study remains the most complete example of a transcriptomics-to-population pipeline in the AD literature.

This approach enables scalable identification of pathway-level concordance between drug-induced perturbations and disease signatures, with the added potential for genotype stratification. Three limitations are particularly important. First, the dominant perturbational reference datasets were generated in immortalised, largely cancer-derived cell lines whose transcriptional responses may not represent neurons, microglia or astrocytes. Second, bulk post-mortem tissue aggregates multiple cell types at a single, terminal disease stage; a signature derived from end stage cortex may not describe the biology of the early symptomatic patients who would be enrolled in a trial. Third, and most fundamentally, transcriptomic reversal is a surrogate whose relationship to clinical outcome has never been prospectively demonstrated in AD.

### 3.2 Network medicine and systems pharmacology

Network medicine formalizes an intuitive concept: disease genes are not randomly distributed within the interactome but instead cluster into modules; a drug is more likely to have therapeutic value if its target lies within or adjacent to such a module (Cheng et al., 2018; Guney et al., 2016). In AD, this has been operationalised through the endophenotype concept: rather than modelling AD as one module, investigators construct separate modules for amyloid, tau, neuroinflammation and vascular endophenotypes and compute drug proximity to each module (Fang et al., 2020).

The most influential application combined this framework with population validation. Fang and colleagues computed network proximity of approved drugs to AD endophenotype modules, identified sildenafil among the top-ranked candidates, and then tested the prediction in a claims database of 7.23 million individuals, reporting a 69% reduction in incident AD among sildenafil exposed individuals relative to comparators over 6 years of follow-up. The team then examined sildenafil in iPSC-derived neurons, observing reduced tau phosphorylation and modulation of neuroinflammatory and neuroplasticity pathways (Fang et al., 2021). A subsequent framework from the same group integrated genome-wide association data, multi-omics and interactome analysis to nominate additional candidates including pioglitazone, with *in vitro* validation in human microglia (Fang et al., 2022). The AlzGPS platform makes the underlying proximity computations broadly accessible within a publicly available resource (Zhou et al., 2021).

Network-based approaches provide mechanistic plausibility grounded in human genetics and PPI topology and can identify polypharmacological candidates that engage several endophenotypes. Network proximity is a topological statement about a static, incomplete graph. Estimates suggest that only a minority of the human interactome has been mapped, and interaction maps are biased toward well-studied proteins—which systematically favours candidates in well-studied pathways. Proximity carries no information about whether a drug reaches the CNS, engages its target at achievable exposures, or acts at the disease stage of interest, although CNS constraints can be imposed as a separate pre-filtering step (Section 5.2). Moreover, the framework also does not model disease trajectories: its modules are inferred from cross-sectional data and treated as static, whereas the pathophysiological process of AD evolves continuously over decades.

### 3.3 Real-world data and pharmacoepidemiology

Pharmacoepidemiology directly applies human population data to drug evaluation. Using EHR and claims databases, together with formal causal inference methods such as target trial emulation, propensity weighting and active-comparator new-user designs, it can assess whether individuals exposed to a candidate drug develop AD at lower rates than comparable unexposed individuals.

Studies of GLP-1 receptor agonists illustrate both the strengths and limitations of this approach. Wang and colleagues emulated seven target trials in a network of 116 million US EHR records, comparing semaglutide against seven antidiabetic comparators, and reported consistently lower rates of first-time AD diagnosis in patients with type 2 diabetes (Wang W. et al., 2024). Zhang and colleagues, using covariate-adjusted Cox models across two large real-world databases, found lower incident AD risk among initiators of GLP-1 receptor agonists and SGLT-2 inhibitors than among DPP-4 inhibitor initiators (hazard ratios ≤0.69 and ≤0.67, respectively; both p < 0.001) (Zhang et al., 2025). Tang and colleagues, using target trial emulation in the OneFlorida+ consortium, reported similar directional findings (Tang et al., 2025). Moreover, Nørgaard and colleagues pooled randomised trial data with Danish national registry records covering 120,054 individuals and reported lower dementia incidence with GLP-1 receptor agonist exposure (Norgaard et al., 2022).

The consistency of these signals across databases, countries and analytical teams was widely read as robust. However, the findings were not uniform. In a UK primary-care cohort from the Clinical Practice Research Datalink using propensity-score overlap weighting, dementia risk did not differ between GLP-1 receptor agonist and DPP-4 inhibitor initiators in the intention-to-treat analysis (hazard ratio 0.95, 95% CI 0.87–1.04); an association emerged only in an as-treated analysis restricted to continuous use (hazard ratio 0.79, 95% CI 0.64–0.97) (Wu et al., 2025). The difference between these estimands is important because treatment persistence and the health status associated with persistence can influence observational effect estimates.

The strength of pharmacoepidemiology is that it evaluates drug associations directly in large human populations with real-world exposure. Its central limitation is that lower incident dementia risk does not establish efficacy in established AD. Such associations may reflect true prevention in an at-risk population, improved metabolic or vascular health acting years before symptom onset, differential prescribing, surveillance bias or residual confounding. These explanations are not mutually exclusive and cannot be distinguished from observational data alone. Prevention therefore remains a hypothesis until tested prospectively.

### 3.4 Multi-omics integration and deep learning

Multi-omics approaches integrate genomic, transcriptomic, proteomic, metabolomic, single-cell and imaging data using graph neural networks and other deep architectures, on the premise that no single data layer captures AD pathophysiology adequately. Cheng and colleagues have described a framework integrating genome, transcriptome, proteome, radiome and phenome data for candidate prioritisation and AD patient stratification (Cheng et al., 2024). Applications of single-nucleus RNA (snRNA) sequencing have identified molecular networks linking disease-associated microglia and astrocytes, with direct implications for which cell type a candidate drug would need to reach (Xu et al., 2021).

Deep models trained on heterogeneous multi-omics data are difficult to interrogate, which matters when the output is intended to justify a clinical trial rather than to make a prediction. Batch effects and cohort heterogeneity limit generalisability across datasets. Very few DL-nominated AD candidates have advanced beyond preclinical evaluation. An adjacent example is DeepDrugDiscovery (Dong et al., 2026), a mechanism-aware AI screening platform that integrates ADMET and BBB penetrability predictions. The platform identified mTOR-independent autophagy enhancers, two of which crossed the BBB, reduced AD-related protein aggregates and improved memory in *Caenorhabditis elegans* and mouse models of AD. This is a strong preclinical demonstration of AI-guided prioritisation, but it illustrates *de novo* drug discovery rather than clinical drug repurposing: the compounds are novel chemical matter without prior human exposure data, and none has entered clinical testing. We include it as a boundary example of a method that could support future repurposing workflows, not as an instance of repurposing itself.

### 3.5 Large language models: a cross-cutting methodological layer

LLMs have been applied to AD repurposing in three modes: synthesis of published literature into ranked candidate lists; reasoning over structured biomedical knowledge graphs; and orchestration of multi-step analytical workflows.

The GPT-4 study illustrates both the utility and the limitations of literature-driven LLM nomination. Yan et al. queried GPT-4 iteratively for the twenty most promising AD repurposing candidates, then tested the top ten for incident AD in two large clinical datasets—Vanderbilt University Medical Center records and the All of Us Research Program. In their meta-analysis, metformin (hazard ratio 0.67, 95% CI 0.55–0.81), simvastatin (0.84, 0.73–0.98) and losartan (0.76, 0.60–0.95) were associated with lower AD risk (Yan et al., 2024). The pipeline is efficient and the validation design is sound. The authors themselves observe, however, that the drugs the model proposed most frequently were not novel repurposing candidates but agents frequently co-mentioned with AD in the literature. An important limitation is that an LLM’s output distribution reflects the training corpus, so candidate frequency tracks publication frequency rather than mechanistic promise.

Knowledge-graph reasoning represents a more distinct methodological application. Wang and colleagues (Wang et al., 2025) compared five repurposing strategies for AD, including a graph neural network approach, a machine learning model over the Alzheimer’s Knowledge Base (AlzKB), and their ESCARGOT framework combining Graph-of-Thoughts LLM reasoning with AlzKB. The LLM-augmented knowledge graph approach outperformed the alternatives on their benchmarks. Here the LLM does not provide evidence itself but rather navigates a structured evidence repository more effectively than embedding-based methods, and this constitutes a genuine methodological contribution.

A third application extends AI nomination to drug combinations. The DeepDrug framework incorporated expert-defined biological knowledge, including long genes, immunological and ageing pathways, and somatic mutation markers associated with AD, into a graph-neural-network framework and proposed a lead five-drug combination (Li et al., 2025). Somatic mutation evidence is particularly relevant because mosaic mutations in genes including ADNP have been identified in AD brain tissue and linked experimentally to tau-related pathology (Ivashko-Pachima et al., 2021). However, the DeepDrug combination itself has not yet been validated experimentally at the combination level.

LLMs can accelerate evidence synthesis, compress literature review and facilitate traversal of knowledge graphs, but they do not constitute an independent evidence domain. An LLM-generated candidate list derives its content from the same published studies that populate the other four domains and treating it as corroborating evidence double-counts that literature. Three further limitations bear on any use of LLM output in a translational pipeline: susceptibility to fluent but unsupported assertion, requiring cross-validation against structured databases; sensitivity of output to prompt formulation, with reproducibility across prompting strategies not yet systematically characterised; and training data cut-offs that render models blind to recent evidence. We suggest that LLM-derived nominations be labelled as exploratory until independently validated within one or more of the four evidence domains.

### 3.6 Validation of computational nominations

The translational value of a computational ranking depends on how that ranking is evaluated. In the 124-study review cited above, 94% of experimental studies performed some form of validation compared with 44% of computational studies, and only 31% of all studies used a validation system intended to reflect AD (Grabowska et al., 2023). Candidate ranking is therefore still frequently treated as an endpoint rather than as the start of a validation process.

Three issues are especially important. First, retrospective evaluation can be circular when positive labels, literature co-mentions or known AD drugs are drawn from the same sources used to train or populate the model. Recovery of familiar drugs can demonstrate internal consistency, but it is a weak test of predictive novelty; a similar problem is evident in literature-driven LLM nomination, where frequently discussed candidates are preferentially recovered (Yan et al., 2024). Second, evaluation sets and negative controls vary widely across studies, so reported performance is difficult to compare between methods. Third, retrospective rediscovery is weaker evidence than a prospectively specified nomination that is subsequently confirmed in an independent experimental or clinical domain.

Computational repurposing studies should therefore report the temporal cut-off and provenance of their training or knowledge sources, define whether evaluation labels could have been present in those sources, include appropriate negative controls or temporally held-out tests where feasible, and state what independent result would falsify the ranking. These requirements do not establish clinical efficacy, but they make the computational step itself auditable before translational resources are committed.

## 4 From nomination to translation: candidates by evidence maturity

As discussed above, different AI methods are suited to addressing different types of questions. Therefore, drug candidates should not be evaluated solely on the strength of a single prediction or by extrapolation from a drug class. Instead, each candidate requires a comprehensive assessment that integrates information about the specific molecule across diverse and independent lines of evidence.

To distinguish mature translational programmes from early-stage hypotheses, we organise candidates into evidence-maturity tiers ranging from clinically falsified agents and drugs undergoing late-stage evaluation to candidates supported by multimodal preclinical evidence, population-level signals or exploratory AI-generated hypotheses. Each candidate is then assessed across six dimensions: computational nomination; human genetic or experimental support; CNS pharmacology; real-world evidence; clinical evidence; and the principal remaining uncertainty. Table 2 presents this systematically. Its six dimensions are descriptive rather than normative, characterising the evidence that currently exists for each molecule; the requirements that we propose should be satisfied before clinical progression are set out separately in Section 5.

**TABLE 2:** Appraisal of leading repurposing candidates in AD by evidence maturity, assessed at the level of the individual molecule.

Panel A. Computational, experimental and pharmacological evidence			
Candidate	Computational nomination	Genetic/ experimental support	CNS pharmacology
Semaglutide (GLP-1 RA)	Not computationally nominated in origin; surfaced by GPT-4 literature synthesis (Yan et al., 2024) and by EHR analyses (Wang et al., 2024b; Zhang et al., 2025)	No GWAS link between GLP-1R signalling and AD risk. Mechanistic rationale (neuroinflammation) proposed, not demonstrated in human brain	Limited CSF exposure reported; central versus peripheral mediation unresolved; no amyloid or tau PET pharmacodynamic assessment in evoke (Cummings et al., 2026b)
Mirodenafil (AR1001; PDE5 inhibitor)	Not Computationally nominated. Programme originated in sponsor preclinical work; conceptually adjacent to sildenafil network evidence (Fang et al., 2021), but developed independently	Preclinical amyloid and memory findings; Phase 2 trial in 210 participants was safe but did not meet co-primary efficacy endpoints (Greeley et al., 2025)	Molecule-specific human CNS exposure remains insufficiently characterised in the public literature
Sildenafil (PDE5 inhibitor)	Endophenotype network proximity to amyloid, tau and vascular modules (Fang et al., 2021)	GWAS-supported vascular endophenotype; iPSC-derived neurons showed reduced tau phosphorylation and neuroinflammatory modulation (Fang et al., 2021)	Crosses blood–brain barrier; dose–response for AD endpoints not characterised and adequate human central target exposure not established (Hainsworth et al., 2023)
Bumetanide (NKCC1 antagonist)	APOE4 genotype-conditioned CMap signature reversal (Taubes et al., 2021) — the most complete precision nomination in AD to date	APOE4 knock-in mouse validation; genotype-matched human cell data; NKCC1/GABA mechanism biologically coherent (Taubes et al., 2021)	Limited brain penetration. Whether therapeutic central concentrations are achievable at tolerated doses is unresolved
Baricitinib (JAK1/2 inhibitor)	DRIAD machine learning nomination from AMP-AD transcriptomics; highest-ranked JAK inhibitor (Rodriguez et al., 2021)	AD GWAS enrichment for immune loci (TREM2, BIN1, CLU); JAK-STAT plausible in microglial activation; preclinical neural culture data	Limited brain permeability has been predicted at recommended clinical doses (Faquetti et al., 2024); NADALS detected baricitinib in CSF, but adequacy for target engagement remains uncertain (Massachusetts General Hospital, 2026)
Metformin, SGLT-2 inhibitors, statins, losartan	GPT-4 synthesis with EHR validation (Yan et al., 2024); SGLT-2i independently nominated within AI combination (Li et al., 2025)	Metformin: AMPK/mTOR, preclinical neuroprotection. SGLT-2i, statins, losartan: mixed and largely indirect	Metformin: limited characterisation for AD endpoints. SGLT-2i: central versus peripheral mediation unresolved
LLM-generated and combination hypotheses	ESCARGOT LLM–knowledge graph reasoning (Wang et al., 2025); DeepDrug multi-drug combination (Li et al., 2025); GPT-4 candidate lists (Yan et al., 2024)	None independent of the published literature from which the nominations derive	Not assessed for individual components or combinations

Panel B. Real-world, clinical and unresolved evidence			
Candidate	Real-world evidence	Clinical evidence	Principal remaining uncertainty
Semaglutide (GLP-1 RA)	Consistent across several databases (Wang et al., 2024b; Zhang et al., 2025; Tang et al., 2025; Norgaard et al., 2022); not uniform — null in ITT analysis of UK CPRD cohort (Wu et al., 2025)	evoke/evoke+ Phase 3, n = 3,808: CDR-SB difference −0.08 (95% CI −0.35 to 0.20) and 0.10 (−0.17–0.38); 104-week efficacy endpoints negative and planned extension discontinued (Cummings et al., 2026b)	Whether a genuine preventive effect exists in metabolically at-risk populations. Untested; requires a prospective prevention trial
Mirodenafil (AR1001; PDE5 inhibitor)	Nonspecific to mirodenafil. Sildenafil claims evidence (Fang et al., 2021) does not transfer between molecules	POLARIS-AD Phase 3 (NCT05531526), early amyloid positive AD; enrolment closed, results unavailable at cut-off (AriBio Co Ltd, 2026)	Phase 3 outcome and adequacy of molecule-specific CNS exposure. The programme is not a direct test of the sildenafil AI nomination
Sildenafil (PDE5 inhibitor)	7.23M insurance claims; ∼69% lower incident AD versus comparator (Fang et al., 2021). Effect size implausibly large for an observational estimate	No AD-specific randomised trial	Magnitude of confounding in the claims estimate. Requires biomarker-stratified Phase 2 with pharmacodynamic endpoints
Bumetanide (NKCC1 antagonist)	Two independent EHR databases, >5M individuals: approximately 35%–75% lower AD prevalence across matched analyses (Taubes et al., 2021)	BumxAD Phase 2 (NCT06052163) registered in biologically confirmed AD; no efficacy results available (Stanford University, 2026)	Therapeutic index: limited CNS exposure and loop-diuretic toxicity may prevent sustained target engagement in older adults
Baricitinib (JAK1/2 inhibitor)	Class- or pathway-level epidemiological support from rheumatoid arthritis populations; no baricitinib-specific AD incidence association identified	NADALS Phase 1/2 basket trial (NCT05189106) completed; summary results posted to ClinicalTrials.gov (Massachusetts General Hospital, 2026)	Interpretation of the observed CSF exposure and the limited change in the primary CCL2 biomarker, and whether broader biomarker effects justify efficacy testing
Metformin, SGLT-2 inhibitors, statins, losartan	Metformin HR 0.67; simvastatin 0.84; losartan 0.76 [25]. Losartan estimates directionally opposed between contributing datasets	MAP Phase 2/3 trial (NCT04098666) in amnestic MCI is ongoing; no completed AD-specific Phase 3 efficacy result (Columbia University, 2026)	Mechanism, disease-stage specificity and sustainable funding for generic compounds
LLM-generated and combination hypotheses	EHR validation performed for three GPT-4 candidates (Yan et al., 2024); reflects publication frequency rather than novel discovery	No trials initiated for any LLM-specific or AI-derived combination hypothesis	Evidentiary standing. Legitimate hypothesis-generating output requiring validation in an independent evidence domain before clinical consideration

The six columns across Panels A and B are descriptive: they record what evidence currently exists for each molecule, not what evidence should be required. They are therefore distinct from the seven normative requirements proposed in Section 5, and the final column, principal remaining uncertainty, is a summary judgement rather than an evidentiary criterion. Candidates are ordered by evidence maturity, from clinically falsified through late-stage evaluation to exploratory hypotheses.

Importantly, evidence generated for one molecule does not necessarily transfer to another molecule of the same class. This applies with particular force to the phosphodiesterase-5 (PDE5) inhibitors, where the network medicine and pharmacoepidemiologic evidence concern sildenafil while the compound in Phase 3 is mirodenafil. Figure 2 translates this principle into a staged decision framework.

**FIGURE 2:**
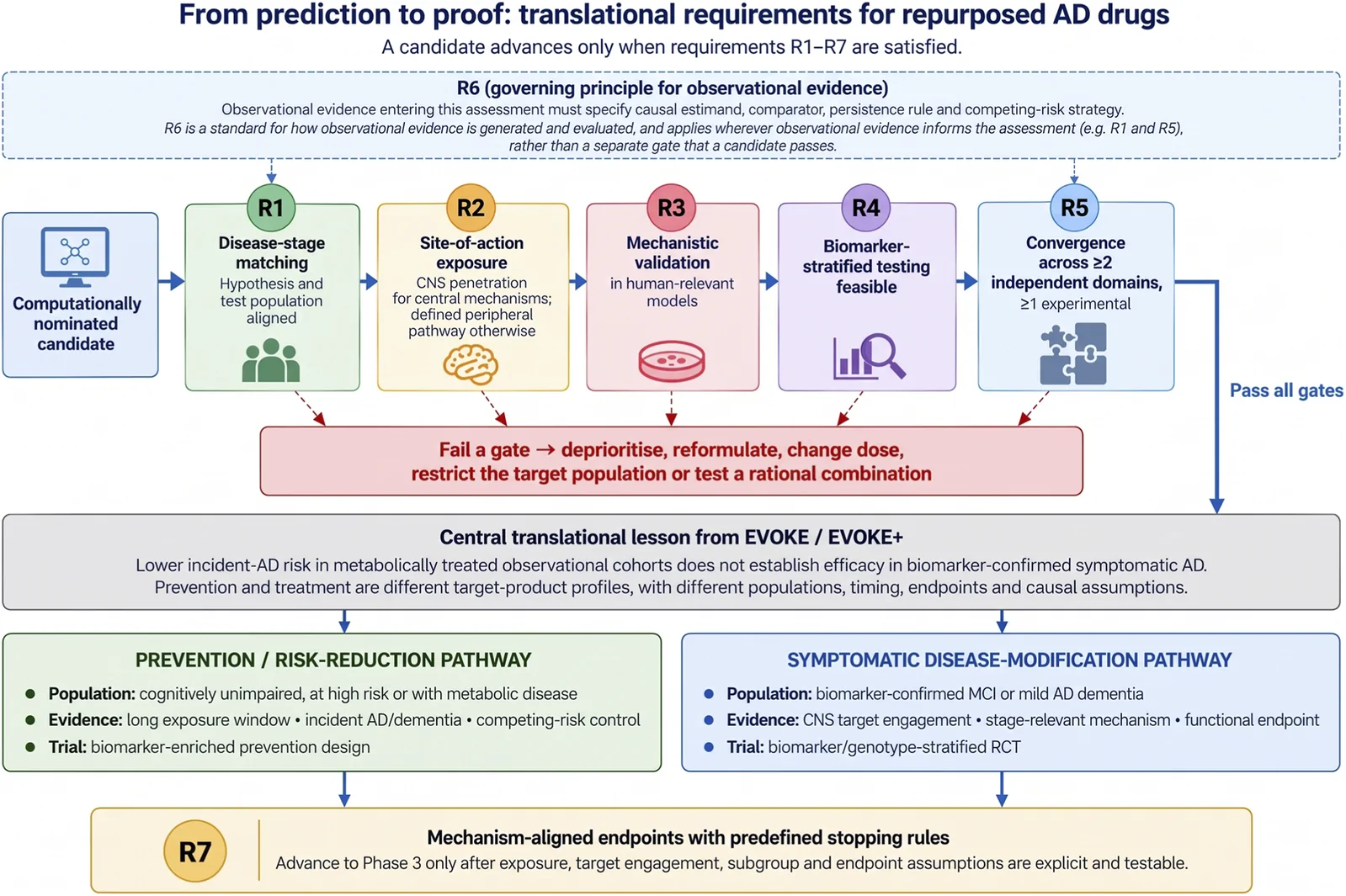
From prediction to proof: translational requirements for repurposed AD drugs. The gates correspond to the seven evidentiary requirements set out in Section 5 and are shown in the same order. A candidate should advance only after disease-stage matching between the observed signal and the intended treatment population (R1), demonstration of adequate exposure and target engagement at the proposed site of action, including blood–brain barrier penetration for central mechanisms (R2), mechanistic validation in human-relevant models (R3), and feasible biomarker-stratified testing (R4), with supporting evidence converging across at least two independent domains of which at least one is experimental (R5). R6 is shown as a governing principle rather than a sequential gate, because it specifies how observational evidence should be generated and evaluated wherever such evidence informs the assessment, particularly at R1 and R5, rather than constituting a hurdle that a candidate passes. Failure at any gate should lead to reprioritization, reformulation, dose modification, restriction of the target population or testing of a rational combination, rather than to progression. Candidates passing all gates proceed to pathway selection: prevention and symptomatic disease modification require distinct target-product profiles and trial designs. R7, mechanism-aligned endpoints with predefined stopping rules, applies at the point of trial design.

### 4.1 Phase 3 negative in early symptomatic AD: semaglutide

The evoke and evoke+ trials provide the most consequential recent falsification of a high-profile AD repurposing hypothesis. Semaglutide was not computationally nominated in origin, so these trials do not directly test whether an AI ranking is correct. They are nevertheless highly informative for the central question of this review because they test the broader inference that convergent observational and mechanistic support is sufficient to justify efficacy expectations in symptomatic AD (Cummings et al., 2026b).

Both trials were well designed and rigorously conducted. Between May 2021 and September 2023, 9981 individuals were screened and 3,808 randomised across the two trials: 1855 in Evoke (semaglutide n = 928, placebo n = 927) and 1953 in Evoke+ (semaglutide n = 976, placebo n = 977). Participants had mild cognitive impairment or mild dementia due to AD with amyloid positivity confirmed by positron emission tomography or cerebrospinal fluid (CSF), a mean age of 72.2 years, and were permitted to continue approved AD therapies including anti-amyloid antibodies. Evoke+ additionally permitted participants with cerebral small vessel disease. The primary endpoint was change in CDR-SB from baseline to week 104 (Cummings et al., 2026b; Scheltens et al., 2026).

Mean CDR-SB change from baseline to week 104 was 2.3 with semaglutide versus 2.3 with placebo in Evoke (estimated difference −0.08, 95% CI −0.35 to 0.20, p = 0.57), and 2.2 versus 2.1 in Evoke+ (estimated difference 0.10, 95% CI −0.17 to 0.38, p = 0.46). Confirmatory secondary endpoints, including the Alzheimer’s Disease Cooperative Study Activities of Daily Living Scale for MCI and time to CDR global worsening, were likewise negative, as were Mini-Mental State Examination and ADAS-Cog outcomes. A pooled analysis showed no delay in progression from MCI to mild AD. Safety was consistent with semaglutide’s established profile; treatment-emergent adverse events occurred in 91.2% of semaglutide and 84.8% of placebo recipients, with five investigator-assessed treatment-related deaths (one semaglutide, four placebo). The week-104 efficacy analyses were negative, and both trials were subsequently discontinued because of the negative clinical outcome (Cummings et al., 2026b).

These results should be interpreted narrowly. What Evoke establishes is that oral semaglutide 14 mg does not slow clinical progression over 2 years in early symptomatic, amyloid-positive AD. What it does not establish is that the observational literature was wrong about what it measured. Those studies examined incident dementia diagnosis in patients with type 2 diabetes over extended exposure--a different population, a different outcome and a different disease stage. The appropriate conclusion is that observational associations with lower incident AD risk do not establish efficacy for treating biomarker-confirmed symptomatic AD. Whether GLP-1 receptor agonists exert a genuine preventive effect in metabolically at-risk populations remains an open question that evoke was not designed to answer and cannot answer.

Two features of the trials constrain mechanistic inference further. CSF biomarkers were collected only in a substudy of 199 participants, and neither amyloid nor tau positron emission tomography was performed for pharmacodynamic assessment (Cummings et al., 2026b). The proportion of evoke+ participants with documented small vessel pathology was 2.8%, which limits what can be concluded about the vascular subgroup the trial was partly designed to interrogate.

A second GLP-1 receptor agonist trial provides a complementary test. In ELAD, 204 participants with mild-to-moderate AD syndrome were randomised to liraglutide or placebo for 52 weeks. The primary FDG-PET outcome, cerebral glucose metabolism, did not differ significantly between groups (difference −0.17, 95% CI −0.39 to 0.06; p = 0.14) (Edison et al., 2026). A secondary ADAS-Exec outcome favoured liraglutide (0.15, 95% CI 0.03–0.28; unadjusted p = 0.01), but CDR-SB and ADCS-ADL did not differ and secondary analyses were not adjusted for multiplicity. ELAD therefore does not provide a second definitive clinical falsification, but it reinforces the need to separate promising class-level biology from a positive prespecified trial endpoint.

### 4.2 Under definitive clinical test: mirodenafil (AR1001)

Mirodenafil (AR1001) is a second-generation PDE5 inhibitor originally approved in South Korea for erectile dysfunction and developed for AD by AriBio. POLARIS-AD (NCT05531526) is a double-blind, randomised, placebo-controlled Phase 3 trial in early AD with confirmed amyloid pathology, with change in CDR-SB at 52 weeks as the primary endpoint. Enrolment has closed, but no Phase 3 efficacy results were available at the review cut-off (AriBio Co Ltd, 2026).

Mirodenafil is frequently discussed alongside the sildenafil network medicine literature, but the two molecules have distinct evidence bases. Mirodenafil’s programme originated in sponsor-led preclinical pharmacology rather than the computational nomination that identified sildenafil. In a 210-participant Phase 2 trial, AR1001 was generally well tolerated, but neither co-primary efficacy endpoint was met after 26 weeks; *post hoc* subgroup and biomarker findings remain hypothesis-generating (Greeley et al., 2025). Claims of greater PDE5 potency or brain penetrance than sildenafil require molecule-specific confirmation and do not permit evidence to be transferred between the two compounds.

Two distinctions are important. First, POLARIS-AD is not a test of the sildenafil hypothesis, and neither a positive nor a negative result should be read as validating or refuting the network medicine prediction that generated it. Second, mirodenafil was not computationally nominated; its development arose from conventional pharmacology and sponsor-led development. The most advanced repurposing programme in AD therefore provides less direct support for the value of computational nomination than is sometimes assumed.

### 4.3 Convergent evidence without definitive trials

Three approved drugs for non-AD indications sildenafil, bumetanide and baricitinib, have subsequently been computationally nominated for repurposing in AD and have progressed beyond prediction to independent experimental, population-level or clinical validation. None has yet demonstrated efficacy in a definitive AD trial, and each retains a specific and identifiable barrier.

#### 4.3.1 Sildenafil

Sildenafil has the most complete evidence chain short of a randomised trial: network proximity to AD endophenotype modules, an association with reduced incident AD in 7.23 million insurance claims records, and mechanistic effects on tau phosphorylation and neuroinflammatory signalling in iPSC-derived neurons (Fang et al., 2021). Sildenafil crosses the BBB, unlike several other candidates considered here, but whether it achieves concentrations sufficient for central target engagement in humans has not been established, and the choice among PDE5 inhibitors for dementia will depend on brain penetration, tolerability in older people and half-life (Hainsworth et al., 2023). Notably, a separate pharmacoepidemiological analysis found no association between PDE5 inhibitor initiation and incident AD (Desai et al., 2022), underscoring that the observational signal is not uniform.

The barrier here is the reliability of the observational estimate. A hazard ratio of approximately 0.31 represents an unusually large observational association and should not be interpreted as an estimate of treatment efficacy. Sildenafil users may differ systematically from comparators in cardiovascular health, general functional status and healthcare engagement in ways that are difficult to measure. Effect sizes of this magnitude from claims data should be treated as hypothesis-generating rather than as estimates of a causal effect. Sildenafil therefore warrants a biomarker-stratified Phase 2 trial incorporating CSF or imaging pharmacodynamic endpoints, but such a trial has not yet been conducted.

#### 4.3.2 Bumetanide

In our assessment, bumetanide remains the most methodologically complete demonstration of AI-enabled precision repurposing in AD. The nomination was genotype-specific rather than generic: APOE4-associated transcriptomic signatures were used to query the CMAP and bumetanide emerged as a signature-reversing candidate. The prediction was then tested in APOE4 knock-in mice and in two independent EHR databases covering more than five million individuals, where bumetanide exposure was associated with approximately 35%–75% lower AD prevalence across matched analyses (Taubes et al., 2021). The proposed mechanism, involving NKCC1 inhibition and subsequent modulation of GABAergic signalling, is biologically plausible.

The remaining barriers are pharmacological and clinical rather than computational. Bumetanide has limited brain penetration, and it remains unresolved whether therapeutically meaningful central concentrations can be achieved at doses tolerated by older adults. Its loop-diuretic effects create risks of volume depletion, electrolyte disturbance, hypotension and falls. A randomised Phase 2 study in biologically confirmed AD, BumxAD (NCT06052163), has been registered to examine safety, tolerability and preliminary clinical and biomarker outcomes (Stanford University, 2026). This trial tests whether an APOE4-conditioned transcriptomic prediction remains viable despite bumetanide’s limited CNS penetration and diuretic toxicity.

#### 4.3.3 Baricitinib

Baricitinib was nominated by DRIAD as the highest-ranked JAK inhibitor (Rodriguez et al., 2021), and the underlying pathway rationale is well supported. We note at the outset that the epidemiological support for this candidate is class- or pathway-level rather than molecule-specific: it derives from observations of anti-inflammatory and JAK-directed therapy in rheumatoid arthritis cohorts, and in our search we identified no baricitinib-specific association with AD incidence. By the molecule-level principle set out above, this is weaker than the population evidence available for sildenafil or bumetanide. The pathway rationale is nonetheless sound: AD genome-wide association signals are enriched for immune loci including TREM2, BIN1 and CLU, and JAK-STAT signalling is a plausible node in microglial inflammatory activation. The candidate was carried into NCT05189106, an open-label, biomarker-driven basket trial at Massachusetts General Hospital enrolling individuals with subjective cognitive decline, MCI, AD or amyotrophic lateral sclerosis. Participants received 2 mg daily for 8 weeks followed by 4 mg daily for 16 weeks (Massachusetts General Hospital, 2026).

The design of that trial is notable for what it prioritised. Its stated aim was to determine whether baricitinib reaches measurable concentrations in CSF and whether inflammatory biomarkers change before efficacy is tested. This ordering is appropriate, particularly because pharmacological analyses suggest limited brain permeability at recommended clinical doses (Faquetti et al., 2024). The study was completed in June 2025, and summary results were posted to ClinicalTrials.gov in June 2026 (Massachusetts General Hospital, 2026). In the AD group (n = 7), mean CSF baricitinib concentration was 1.244 nmol/L after the 2-mg phase and 2.469 nmol/L after the 4-mg phase (Massachusetts General Hospital, 2026). The registry also reports CCL2 and other inflammatory biomarkers, but the small, open-label dataset does not establish therapeutically adequate target engagement or clinical benefit. A peer-reviewed report had not been identified by our review cutoff.

### 4.4 Population signals requiring mechanistic and pharmacokinetic groundwork

A fourth group has population-level signals without the mechanistic and pharmacokinetic groundwork required for Phase 3 commitment.

Metformin is supported by the LLM-plus-EHR meta-analysis described above (hazard ratio 0.67) (Yan et al., 2024) and by extensive preclinical literature involving AMPK/mTOR signalling and metabolic inflammation. The Metformin in Alzheimer’s Dementia Prevention trial (MAP; NCT04098666) is a multicentre Phase 2/3 randomised trial of extended-release metformin in 326 adults with amnestic MCI who do not have diabetes (Columbia University, 2026). Its prevention-oriented design, with cognitive, imaging and biomarker outcomes, is better matched to the metabolic-risk hypothesis than a trial in established dementia. The continuing difficulty of financing large trials of generic metformin, including the broader Targeting Aging with Metformin (TAME) (Barzilai et al., 2016) initiative, nevertheless illustrates the structural disadvantage faced by off-patent drugs.

SGLT-2 inhibitors have accumulated pharmacoepidemiological support from several independent groups (Zhang et al., 2025; Tang et al., 2025) and were independently nominated within an AI-derived combination (Li et al., 2025). Central exposure is less clearly limiting for this class than is sometimes assumed: SGLT2 expression has been documented in the CNS, and proposed mechanisms include reduced oxidative stress and neuroinflammation, decreased amyloid burden, enhanced neuronal plasticity and improved cerebral glucose utilisation (Mei et al., 2024). What remains unresolved is whether the observed epidemiological associations are mediated centrally or through systemic glycaemic control, blood pressure, renal function and peripheral inflammation (Zhang et al., 2025; Tang et al., 2025). Distinguishing these is a prerequisite for rational trial design rather than a detail, since the two accounts imply different target populations and different pharmacodynamic endpoints.

Statins and angiotensin receptor blockers occupy a similar position with an additional caution. In the ChatGPT-4 validation study, losartan showed an overall protective association in the pooled meta-analysis, although the effect estimates differed in direction between the two contributing datasets (Yan et al., 2024). This inconsistency reduces confidence in the robustness and generalisability of the pooled association and suggests that the apparent protective effect may be sensitive to cohort composition, residual confounding or differences in exposure and outcome ascertainment between databases. The losartan signal should therefore be regarded as hypothesis-generating rather than as strong evidence for an AD-protective effect.

For all agents in this group, three requirements should be met before clinical progression: mechanistic validation in human CNS-relevant models; demonstration of adequate exposure at the intended site of action or a clearly defined peripheral mechanism, and prospective testing in a population matched to the disease stage in which the signal arose.

### 4.5 Exploratory: LLM-generated and combination hypotheses

Finally, candidates arising from LLM synthesis and AI-derived combinations should be classified as exploratory. This is not a dismissal but a statement about evidentiary standing. The ChatGPT nominations reflected publication frequency rather than novel mechanistic insight (Yan et al., 2024); the ESCARGOT framework demonstrated improved knowledge graph traversal rather than new biology (Wang et al., 2025); and the DeepDrug combination has not been tested experimentally as a combination (Li et al., 2025). Each is a legitimate hypothesis-generating output. None has yet been subjected to validation within an independent evidence domain, and none should compete for clinical resources with candidates that have.

## 5 Translational requirements: what computational nomination alone cannot establish

A recent expert-led prioritisation pipeline for a planned multi-arm AD platform trial ranked repurposed compounds according to biological plausibility, preclinical efficacy, safety and trial feasibility (Iqbal et al., 2026). Our framework addresses a complementary question: what independent evidence should be required after computational nomination before a specific molecule progresses to clinical testing. We propose seven evidentiary requirements (R1–R7) for this progression. Section 5.8 addresses a separate implementation constraint created by weak commercial incentives and is not an eighth evidentiary requirement.

### 5.1 R1. Disease-stage matching

A central lesson from the evoke trials is that an association observed in one population may not translate into clinical benefit in another. The pharmacoepidemiological evidence for GLP-1 receptor agonists was generated in patients with type 2 diabetes, over years of exposure, with incident dementia diagnosis as the outcome. The trial was conducted in amyloid-positive individuals with established cognitive symptoms, over 2 years, with rate of clinical progression as the outcome. These are different questions about different populations at different points in the disease trajectory, and the extent to which a prevention-stage association would predict efficacy in symptomatic disease was uncertain.

We therefore suggest that repurposing programmes should specify the disease stage at which the hypothesis applied, e.g., preclinical, MCI, or mild dementia, defined by amyloid, tau and neurodegeneration biomarkers, and should generate their computational evidence from data representing that stage. A hypothesis derived from prevention-stage epidemiology should be tested in a prevention trial. If prevention trials are constrained by duration and cost, these limitations should be addressed directly rather than testing the hypothesis in a population that does not match the original signal.

### 5.2 R2. Site-of-action pharmacology as a mandatory filter

Any drug nominated for AD must demonstrate pharmacologically adequate exposure at its proposed site of action. For a central mechanism, this requires blood–brain barrier penetration at clinically achievable doses, sufficient unbound brain exposure for target engagement, and ideally CSF or imaging evidence of engagement. For a primarily peripheral mechanism—such as metabolic, vascular, renal or immune modulation—brain penetration is not itself mandatory, but systemic exposure and the causal pathway linking peripheral target engagement to AD-relevant biology should be demonstrated.

The candidates reviewed above show how often this is neglected. The evoke programme included a CSF substudy in 199 participants, but the trials were not designed to distinguish central from peripheral mediation and did not include amyloid or tau PET pharmacodynamic assessment (Cummings et al., 2026b). Baricitinib’s brain permeability at recommended doses has been questioned (Faquetti et al., 2024), although NADALS has now demonstrated measurable CSF exposure (Massachusetts General Hospital, 2026). Bumetanide’s central exposure at tolerable diuretic doses remains unestablished (Taubes et al., 2021). In each case, computational or observational nomination alone is silent on whether sufficient drug reaches and engages the intended site of action.

This gap is beginning to be addressed within network-based approaches. Akgüller and colleagues constructed a CNS-focused network medicine framework in which machine-learning-based blood–brain barrier penetration prediction was applied as a pre-filter rather than a *post hoc* check, reducing 24,474 compounds from the Drug–Gene Interaction Database to 8,247 CNS-relevant candidates before network scoring, with modality-specific tractability assessment applied separately to small molecules, peptides and biologics (Akguller et al., 2025). The authors offered recovery of memantine and donepezil among the top-ranked candidates as a face-validity check; as discussed in Section 3.6, rediscovery of well-annotated drugs is an internal benchmark rather than independent validation. The authors were explicit that the resulting predictions remain hypothesis-generating and require systematic experimental validation rather than constituting therapeutic recommendations.

We propose generalising this ordering across all four evidence domains, rather than applying it within network-based approaches alone. Transcriptomic signature matching, pharmacoepidemiological screening and multi-omics ranking are each often conducted without explicit site-of-action exposure constraints, and each can nominate candidates whose pharmacology later proves limiting. Applying a pharmacokinetic and pharmacodynamic filter before commissioning experimental validation matters economically as well as scientifically, since it is considerably cheaper to exclude a candidate on exposure or target-engagement grounds than to discover the problem in Phase 2. NADALS illustrates the value of measuring CSF exposure and pharmacodynamic biomarkers before efficacy when a central mechanism is proposed; its posted results also show why measurable exposure should not automatically be equated with target engagement (Massachusetts General Hospital, 2026).

### 5.3 R3. Mechanistic validation in human models

Of 124 AD repurposing studies published between 2012 and 2022, 61% included some validation, but only 31% used a system intended to reflect AD. Among the AD-specific validations, 25 studies used animal models, seven used cell-based models, five used real-world data and three were clinical trials (Grabowska et al., 2023). Many computational studies therefore still stop at candidate ranking without human mechanistic or clinical validation.

Human iPSC-derived neurons, microglia, astrocytes and cerebral organoids now provide an intermediate tier of evidence between computation and clinical trial that is human, mechanistic and tractable. The sildenafil programme used iPSC-derived neurons to demonstrate effects on tau phosphorylation and neuroinflammatory signalling (Fang et al., 2021); the bumetanide programme used APOE4 knock-in mice and genotype-matched human cells (Taubes et al., 2021).

Human iPSC-derived neurons, microglia and cerebral organoids are increasingly used for mechanistic study and therapeutic screening in AD (Sainz et al., 2024; Kumari et al., 2026), and in our view evidence of the predicted molecular effect in a human, disease relevant model should be the default expectation before a computationally nominated candidate enters clinical planning. We would not regard this as an absolute requirement. Where the mechanism cannot be adequately modelled in available cell systems, or where a compound already has extensive human exposure data and well-characterised target engagement in another indication, alternative forms of experimental evidence may reasonably suffice. At least one line of support should therefore be experimental rather than purely observational. Human cellular models provide the most informative evidence, but other well-justified experimental approaches may also be acceptable. The limitations of these systems also warrant acknowledgement: iPSC-derived neurons retain a developmentally immature phenotype, do not recapitulate the ageing brain in which sporadic AD arises, and model a disease of decades over weeks in culture. They narrow the gap between computational prediction and clinical trial without closing it.

### 5.4 R4. Biomarker-stratified clinical testing

Most AI repurposing predictions are generated from biomarker-undefined populations: EHR diagnostic codes of variable accuracy, or post-mortem tissue collected years after the relevant therapeutic window. They must nonetheless be tested in biomarker-confirmed populations, and the resulting mismatch is a recurrent source of failure.

The infrastructure for closing this gap has improved substantially. In May 2025, the US Food and Drug Administration (FDA) cleared the Lumipulse G plasma pTau217/beta-amyloid 1-42 ratio to aid identification of amyloid pathology in symptomatic adults evaluated in specialised care. In the supporting study, 91.7% of positive results and 97.3% of negative results agreed with amyloid PET or CSF assessments, although indeterminate results and false classifications remain clinically important (US Food and Drug Administration, 2025). Blood biomarkers do not replace clinical evaluation or confirmatory testing, but they can reduce the cost and burden of screening for biomarker-enriched Phase 2 trials.

Therefore, trial designs should exploit this improved biomarker infrastructure to enrich for biologically relevant patient populations. Basket and platform designs that select participants on the biological feature the drug is hypothesised to modify, as NCT05189106 did with CSF inflammatory markers, allow several computationally nominated candidates to be evaluated against a shared control infrastructure, at a cost per candidate that a non-commercial sponsor can plausibly bear.

### 5.5 R5. Convergence of independent evidence

No single computational method or evidence stream can provide sufficient grounds for clinical trials, and the strength of a computational prediction cannot substitute for the breadth of evidence supporting that prediction. The pattern across the candidates reviewed here supports this conclusion. Sildenafil and bumetanide combine computational nomination with experimental validation and molecule-specific population evidence, whereas baricitinib combines computational nomination and mechanistic rationale with pathway-level epidemiological support and a prospective CNS target-engagement study. By contrast, semaglutide entered definitive clinical testing with strong population-level evidence but comparatively limited molecule-specific support in several other translational domains: no genetic support linking GLP-1 receptor signalling to AD risk, no demonstration of adequate CNS exposure, and no human mechanistic validation at the time the Phase 3 programme was launched.

We propose making convergence across independent evidence domains an explicit criterion for progression to clinical testing. Progression to Phase 2 should require supporting evidence from at least two independent domains, of which at least one must be experimental rather than observational, together with satisfactory site-of-action pharmacology. Candidates that do not yet meet these criteria should not be considered disproven, but they require additional evidence before progression to clinical testing.

### 5.6 R6. Causal inference with explicit boundary conditions

Target trial emulation, active-comparator new-user designs, propensity weighting and negative-control analyses can reduce bias in observational studies, but they cannot remove unmeasured confounding or repair a mismatch between the observed and intended treatment populations. Real-world evidence should therefore be used to define the most plausible population, exposure window and outcome for prospective testing, not to stand in for that testing. Each analysis should pre-specify its causal estimand, comparator, treatment persistence rule and competing-risk strategy, and should report sensitivity to alternative definitions (Wang W. et al., 2024; Zhang et al., 2025; Tang et al., 2025; Wu et al., 2025; Norgaard et al., 2022).

### 5.7 R7. Mechanism-aligned endpoints and stopping rules

Early-phase repurposing trials should first establish exposure and target engagement, then ask whether the expected downstream biology changes. A cognition-only Phase 2 design can be inconclusive when the drug does not reach the brain, fails to engage its target or acts only in a biomarker-defined subgroup. Conversely, a negative site-of-action pharmacokinetic or pharmacodynamic result can terminate an implausible programme before an expensive efficacy trial. The NADALS Baricitinib design illustrates the correct sequence: measure CSF exposure and inflammatory biomarker changes before interpreting clinical outcomes (Massachusetts General Hospital, 2026). Pre-specified early-stopping criteria should be linked to the proposed mechanism rather than to optimism about the candidate.

### 5.8 Implementation constraint: commercial incentives and the off-patent problem

A final constraint is structural rather than scientific and cannot be resolved through methodological rigour alone. Repurposed generic drugs lack patent exclusivity, and the sponsor who funds a definitive Phase 3 trial captures little of the resulting value (Pushpakom et al., 2019; Cummings et al., 2025). The consequences are visible throughout this review.

The long development pathway of TAME illustrates the difficulty of financing large trials of inexpensive generic drugs such as metformin. Bumetanide has one of the most complete precision-repurposing evidence chains in the field, yet prospective testing was delayed for several years. The registered BumxAD Phase 2 study has begun to address this gap (Stanford University, 2026), but still relies on non-commercial trial infrastructure. Meanwhile, the repurposing programme that reached Phase 3 across an international site network is mirodenafil, a proprietary compound with a commercial sponsor. The distribution of clinical investment across the candidates reviewed here tracks intellectual property status at least as closely as it tracks evidence quality.

Addressing this problem will require changes in funding and incentive structures beyond individual research programmes. It requires sustained non-commercial funding for late-phase trials of off-patent agents through national research agencies and disease-focused foundations, and it may require regulatory mechanisms, data exclusivity for new indications, or public-interest trial funding, that alter the return on repurposing a generic. Absent such mechanisms, AI-enabled repurposing will continue to generate candidates that the field cannot afford to test, and the most efficient therapeutic opportunities in AD will remain the least likely to be evaluated.

## 6 Future perspectives

### 6.1 Spatially resolved and cell-type-specific nomination

Spatial transcriptomics is an important near-term development for repurposing. Bulk and even single-cell approaches lose the anatomical context in which AD pathology develops, e.g., the plaque-adjacent microenvironment, laminar cortical organisation, regionally selective vulnerability. Spatially resolved expression integrated with single-cell atlases such as SEA-AD allows candidate nomination to be conditioned on cell type and location simultaneously (Xu et al., 2021; He et al., 2024). A drug predicted to modulate disease-associated microglia in plaque-proximal cortex is a substantially more specific and more testable hypothesis than one predicted to reverse a bulk cortical signature.

### 6.2 Structure-informed repurposing

Structure prediction could help assess target engagement before experimental validation. AlphaFold 3 predicts the joint structure of proteins, nucleic acids, small molecules and ions using a diffusion-based architecture, achieving accuracy for protein-ligand complexes that exceeds conventional docking, which in principle permits computational assessment of whether an approved compound can engage a network-nominated target before experimental resources are committed (Abramson et al., 2024; Fang et al., 2025). Realising this in AD requires systematic application to disease relevant targets and critically prospective validation against experimental binding data. We note again that such studies were not yet available at the time of writing; this is a stated opportunity rather than an established workflow.

### 6.3 Genotype-, endotype- and sex-informed trial design

The bumetanide programme showed that genotype-conditioned nomination is feasible and that it produces sharper hypotheses than population-average approaches (Taubes et al., 2021). Extending this approach would require computational predictions within strata defined by APOE genotype, amyloid and tau status, and inflammatory or vascular endotypes. Trial eligibility should then match the specific stratum in which the prediction was generated. AI methods are well suited to defining such strata, but a major limitation is that clinical trials are rarely designed to test them. Moreover, sex should also be considered explicitly in precision repurposing and trial design. Reanalysis of a randomised prodromal AD trial of davunetide, an ADNP-derived neuroprotective peptide, identified sex-dependent cognitive responses, providing an example of an investigational intervention whose apparent cognitive effects differed between men and women (Gozes et al., 2024). Davunetide is neither a repurposed drug nor an computationally nominated candidate; it is cited here only as an example of a sex-dependent treatment signal. Recent analyses of anti-amyloid therapies similarly suggest sex-related differences in treatment response, although these findings require prospective confirmation (Andrews et al., 2025; Lim et al., 2025). Computational nomination and subsequent clinical testing should therefore consider sex alongside genotype, biomarker status and disease endotype when there is a plausible biological or pharmacological basis for differential efficacy or safety.

### 6.4 Combination therapy

AD’s multi-pathway architecture argues for combination therapy, and network methods can in principle identify combinations whose targets jointly cover several disease modules with limited overlap (Li et al., 2025; Cheng et al., 2019). Computational methods have a clear advantage over empirical approaches in this setting because they can prioritise promising combinations from a search space that is too large for exhaustive experimental testing. A key prerequisite is experimental validation of specific combinations in human neural models. Such validation remains very limited, and the computationally nominated combinations discussed here have not yet been tested in these models.

### 6.5 Imminent readouts

Three programmes will materially inform the framework proposed here. POLARIS-AD (NCT05531526) will provide Phase 3 evidence for mirodenafil in early AD (AriBio Co Ltd, 2026). NADALS (NCT05189106) has demonstrated measurable baricitinib concentrations in CSF in a small open-label cohort, while peer-reviewed interpretation of the full biomarker profile remains awaited (Massachusetts General Hospital, 2026). BumxAD (NCT06052163) will test whether the APOE4-conditioned bumetanide hypothesis is clinically feasible despite limited CNS exposure and diuretic toxicity (Stanford University, 2026).

Each result must be interpreted at the molecule and programme level. A positive POLARIS-AD result would validate mirodenafil, not the sildenafil network prediction. NADALS supports CNS exposure to baricitinib but does not yet establish adequate target engagement or efficacy, leaving open both the molecule-level question and the broader JAK-STAT hypothesis. BumxAD will primarily clarify tolerability, exposure and biomarker response rather than provide definitive efficacy. These distinctions prevent trial outcomes from being used as broad verdicts on AI-enabled repurposing.

## 7 Conclusion

AI has changed how repurposing candidates for AD are identified. Transcriptomic signature matching, network-based systems pharmacology, pharmacoepidemiology and multi-omics DL have each contributed a distinct kind of evidence, and LLMs have added a cross-cutting capacity for literature synthesis and knowledge graph reasoning. Repurposed agents now constitute a third of the AD pipeline and nearly half of Phase 3 agents (Cummings et al., 2026a).

In our review through July 2026, no computationally nominated repurposed candidate had demonstrated efficacy in a definitive AD trial. The evoke and evoke+ trials found no clinical benefit from semaglutide (Cummings et al., 2026b), while ELAD did not meet its primary FDG-PET endpoint for liraglutide (Edison et al., 2026). Neither programme was computationally nominated in origin, so these trials should not be interpreted as direct falsifications of AI. Their value for this review is different: they show that convergent observational, mechanistic or expert prioritisation does not establish efficacy in symptomatic AD and that the population and endpoint used for clinical testing must match the hypothesis being advanced.

The immediate translational bottleneck is not algorithmic performance alone, but the absence of agreed evidentiary standards for acting on computational outputs. We propose seven requirements: R1, disease-stage matching between the observed signal and the treated population; R2, adequate exposure and target engagement at the proposed site of action; R3, mechanistic validation in human-relevant models; R4, biomarker-stratified clinical testing; R5, convergence across independent evidence domains; R6, causal-inference analyses with explicit boundary conditions; and R7, mechanism-aligned endpoints with predefined early stopping rules. The most informative current translational programmes, sildenafil, bumetanide and baricitinib, each satisfy several of these criteria while retaining at least one identifiable gap. Sildenafil lacks a prospective biomarker-stratified trial; bumetanide must demonstrate a workable therapeutic index in BumxAD; and baricitinib has demonstrated measurable CSF exposure but still requires convincing target-engagement and efficacy evidence. In each case the missing evidence is specific and testable.

Computational productivity should not be equated with clinical progress. At present, the strongest claim is that AI has improved the prioritisation of hypotheses for human testing. However, turning those hypotheses into treatments will depend on rigorous translational science and fit-for-purpose funding.
